# A phase 1 evaluation of the pharmacokinetic/pharmacodynamic interaction of the antimalarial agents KAE609 and piperaquine (PPQ)

**DOI:** 10.1186/1475-2875-13-S1-P85

**Published:** 2014-09-22

**Authors:** Daniel S Stein, Jay Prakash Jain, Jason Lickliter, Michael Kangas, Surendra Machineni, Paul Griffin, Gilbert Lefevre

**Affiliations:** 1 Novartis Pharmaceuticals Corporation, East Hanover, NJ, USA; 2 Novartis Healthcare Private Limited, Hyderabad, India; 3 Nucleus Network Limited, Melbourne, Victoria, Australia; 4 Novartis Pharma AG, Basel, Switzerland; 5 Q-Pharm Pty Limited, Brisbane, Queensland, Australia

## Background

KAE609, a spiroindolone, represents a new class of potent, fast-acting, schizonticidal antimalarials. Antimalarial combination treatment is recommended to minimize the potential emergence of resistance on clinical use. Piperaquine (PPQ) is a marketed, long acting antimalarial used in combination with short-acting artemisinins. As both KAE609 and PPQ are CYP3A4 substrates and inhibitors based on *in vitro* or clinical reports, a two way interaction was hypothesized. The potential for both agents to affect the QT interval was also assessed.

## Materials and methods

This was an open-label, parallel-group, single-dose study in healthy volunteers randomized to four parallel dosing arms for five cohorts (2:2:2:2:1) of 75 mg KAE609 plus 320 mg PPQ, 25 mg KAE609 plus 1280 mg PPQ, 25 mg KAE609 alone, 320 mg PPQ alone or 1280 mg PPQ alone. Triplicate ECGs were done over the first 24 hours after dosing, with single ECGs at other time points. Routine safety and pharmacokinetic assessments were carried out up to 89 and 61 days respectively.

## Results

Of the 110 healthy male subjects recruited, 99 completed the study. Co-administration of PPQ had no overall effect on exposure to KAE609, although 1280 mg PPQ decreased KAE609 C_max_ by 17%. 25 mg KAE609 plus 1280 mg PPQ showed a 32% increase, while 75 mg KAE609 plus 320 mg PPQ showed a 14% reduction in PPQ AUC_inf_; the reasons for this are unclear. Mean changes from baseline in QTcF and QTcB with PPQ were consistent with the known effects of PPQ on QTc interval. PPQ but not KAE609 exposure correlated with QTc increase. Also, KAE609 did not affect the PPQ exposure-QTc relationship. The QTcF effect for PPQ (mean maximal change from baseline LS estimate of difference 7.47 msec; 90% CI 3.55, 11.4) was consistent with a positive thorough QT study (ICHE14, mean maximal effect ≥5 msec and upper 95% CI ≥10 msec). No subject had a QTcF or QTcB >500 msec. Most adverse events (AEs) reported were mild; upper respiratory tract infections, headache, diarrhea and oropharyngeal pain were most commonly reported. There were no deaths, serious AEs or severe AEs.

## Conclusions

PPQ and KAE609 co-administration had no clinically relevant effect on exposure to either agent. KAE609 had no effect and did not potentiate the known effects of PPQ on cardiac conduction. Both drugs administered alone or in combination were well tolerated.

